# Change in Magnetic Anisotropy at the Surface and in the Bulk of FINEMET Induced by Swift Heavy Ion Irradiation

**DOI:** 10.3390/nano12121962

**Published:** 2022-06-08

**Authors:** Ernő Kuzmann, Sándor Stichleutner, Libor Machala, Jiří Pechoušek, René Vondrášek, David Smrčka, Lukáš Kouřil, Zoltán Homonnay, Michael I. Oshtrakh, András Mozzolai, Vladimir A. Skuratov, Mátyás Kudor, Bence Herczeg, Lajos Károly Varga

**Affiliations:** 1 Institute of Chemistry, Eötvös Loránd University, Pázmány Péter Sétány 1/A, 1117 Budapest, Hungary; stichleutner.sandor@ek-cer.hu (S.S.); homonnay.zoltan@ttk.elte.hu (Z.H.); mozolaiandras@gmail.com (A.M.); kudor.matyas@gmail.com (M.K.); hercbence@gmail.com (B.H.); 2 Centre for Energy Research, Konkoly-Thege Miklós út 29-33, 1121 Budapest, Hungary; 3 Department of Experimental Physics, Faculty of Science, Palacký University Olomouc, 17. listopadu 1192/12, 771 46 Olomouc, Czech Republic; jiri.pechousek@upol.cz (J.P.); rene.vondrasek01@upol.cz (R.V.); david.smrcka@upol.cz (D.S.); lukas.kouril@upol.cz (L.K.); 4 Department of Experimental Physics, Institute of Physics and Technology, Ural Federal University, 620002 Ekaterinburg, Russia; oshtrakh@gmail.com; 5 Flerov Laboratory of Nuclear Reactions, Joint Institute for Nuclear Research, 141980 Dubna, Russia; skuratov@jinr.ru; 6 Institute of Nuclear Physics and Engineering, National Research Nuclear University MEPhI, 115409 Moscow, Russia; 7 Department of Nuclear Physics, Dubna State University, 141980 Dubna, Russia; 8 Wigner Research Centre for Physics, Konkoly-Thege Miklós út 29-33, 1121 Budapest, Hungary; varga.lajos@wigner.mta.hu

**Keywords:** FINEMET, swift heavy ion irradiation, ^57^Fe transmission and conversion electron Mössbauer spectroscopy, magnetic anisotropy

## Abstract

^57^Fe transmission and conversion electron Mössbauer spectroscopy as well as XRD were used to study the effect of swift heavy ion irradiation on stress-annealed FINEMET samples with a composition of Fe_73.5_Si_13.5_Nb_3_B_9_Cu_1_. The XRD of the samples indicated changes neither in the crystal structure nor in the texture of irradiated ribbons as compared to those of non-irradiated ones. However, changes in the magnetic anisotropy both in the bulk as well as at the surface of the FINEMET alloy ribbons irradiated by 160 MeV ^132^Xe ions with a fluence of 10^13^ ion cm^−2^ were revealed via the decrease in relative areas of the second and fifth lines of the magnetic sextets in the corresponding Mössbauer spectra. The irradiation-induced change in the magnetic anisotropy in the bulk was found to be similar or somewhat higher than that at the surface. The results are discussed in terms of the defects produced by irradiation and corresponding changes in the orientation of spins depending on the direction of the stress generated around these defects.

## 1. Introduction

Unveiling the subtle correlation that links structural ordering and magnetic properties in ferromagnetic amorphous alloys, especially those encompassing outstanding soft magnetic behavior, represents one of the very important areas of research in material science for chemists, physicists, and engineers [1,2,3,4]. These materials can be applied as efficient flux multipliers in a large variety of devices, including transformers, generators, and engines, to be used in the generation, distribution, and conversion of electrical energy [5,6,7,8,9]. From the early discovery [10] of the first soft magnetic amorphous alloy FINEMET; consisting of Fe-Cu-Si-M-B where M can be Nb, Ta, Mo, Zr, W, etc.; the excellent soft magnetism has been shown to occur in a large number of related synthetically engineered amorphous and nanocrystalline alloys [11,12,13,14]. When these Fe-based amorphous alloys were subjected to appropriate annealing, nanoscale grains were formed, embedded in amorphous matrix, resulting in favorable soft magnetic properties [15]. The soft magnetic properties are determined, among other parameters, by the composition of the Fe-based alloy, the grain size, and the volume fraction, as well as by the fine structure of the nanocrystalline Fe-Si grains. Outstanding soft magnetic properties, such as high permeability and low coercive force combined with high magnetic flux density, were obtained with the composition of Fe_73.5_Si_13.5_Nb_3_B_9_Cu_1_-nanostructured FINEMET, which is the most frequently studied FINEMET material, achieved by isothermal heat treatment at 550 °C [16,17,18,19,20,21,22,23,24]. 

The amorphous soft magnetic materials are mostly obtained as thin ribbons through rapid solidification. By a cooling rate in the order of 10^5^–10^6^ K s^–1^ it is possible to overcool the alloy through the glass transition temperature to achieve the amorphous state. The early stage of the primary crystallization of Fe_73.5_Si_13.5_Nb_3_B_9_Cu_1_ FINEMET involves Cu clustering that leads to a desirable nanoscale microstructure. Copper has an important role in the heterogeneous nucleation at the crystallization of α-FeSi. Niobium, with its large atomic radius, inhibits particle growth, and boron, as a non-metal, acts as an amorphizer in the system. Cu promotes the formation of nanocrystals [25,26]. By annealing FINEMET at 550 °C, which results in optimal soft magnetic properties, the microstructure is composed of DO3 type Fe-Si nanocrystals, surrounded by retained amorphous regions [26]. The grain size in the nanocrystalline phase obtained is smaller than the ferromagnetic exchange length. The extreme soft magnetic properties of the optimally annealed FINEMET can be understood in the framework of the random anisotropy model [27,28], since the magneto-crystalline anisotropy is averaged over the many small grains. In several other cases, by examining materials other than the optimal (Fe_73.5_Si_13.5_Nb_3_B_9_Cu_1_) composition, attempts were made to find even better magnetic properties [11,12].

FINEMET materials have been investigated using various methods, including XRD, TEM, SEM, HRTEM, EXAFS, AFM, SANS, DSC, mechanical tests, and magnetic methods [11,14,20,24,25,26,28,29,30,31,32,33,34], as well as by Mössbauer spectroscopy [35,36,37,38]. ^57^Fe Mössbauer spectroscopy is a useful tool for the investigation of amorphous and nanostructured alloys because the ^57^Fe hyperfine interactions are measured, i.e., the electrical monopole and quadrupole interactions as well as magnetic dipole interactions. This method offers a unique possibility to get information about the microenvironment of the Mössbauer nucleus (^57^Fe in this case) [39,40,41]. Mössbauer spectroscopy has already been applied successfully many times to study magnetic and structural properties as well as the crystallization processes in FINEMET alloys (see, e.g., [35,36,37,38,42,43,44,45,46,47,48,49]).

The soft magnetic properties of FINEMET can be further improved by inducing magnetic anisotropy using the application of a magnetic field [50,51] or tensile stress [6,11,52,53,54,55,56,57] during annealing. For nanocrystalline FINEMET, the stress-induced anisotropy is two orders of magnitude higher than the one induced by transverse field annealing [21]. Under stress, the anisotropy induced in the strip develops transversely. Competitive explanations for the mechanism of the stress-induced anisotropy were given by the tensile back-stress theory by Herzer [53] and by the model of atomic pair ordering adapted by Hofmann and Kronmüller [54].

Mössbauer spectroscopic measurements can be advantageously used to follow the formation and change of induced anisotropy in FINEMET alloys. The magnetic anisotropy can be characterized by measuring the relative areas of the second and fifth lines of the Mössbauer sextets (*A*_2,5_), compared to those of the first and sixth lines (*A*_1,6_). This relative area is determined by the angle *θ* included by the directions of magnetic moment and γ-rays according to the formula [40,41]:

*A*_2,5_/*A*_1,6_ = 4 sin^2^*θ*/(3(1 + cos^2^*θ*)) (1)

In the case of stress-field-annealed FINEMET ribbons, the change in the relative areas of the second and fifth lines in the Mössbauer spectra indicated significant variation in the magnetic anisotropy due to the different annealing procedures. A correlation has been established between the permeability and the magnetic anisotropy of the annealed FINEMET samples [48].

Another excellent method to alter the magnetic anisotropy in amorphous and nanocrystalline ribbons is irradiation by swift heavy ions [58]. It was also established that amorphous alloys, compared to crystalline ones, have a very high resistance to radiation damage, so FINEMET can be used in accelerator technology to improve the performance of RF-cavities [59]. In the case of metal-metalloid amorphous alloys irradiated by 225 MeV ^40^Ar ions and 120 MeV ^132^Xe ions, Kuzmann and Spirov (1986) showed that the most striking effect of swift heavy ion irradiation is that the magnetic anisotropy significantly decreases with a change in spin orientation around the radiation-induced defects. This effect increased with the fluence of irradiation. In the case of as-quenched amorphous FINEMET alloys, irradiation-induced changes in the direction of magnetization have already been experienced [47,60,61,62,63]. A coercivity decrease, associated with the annihilation of random stress, was observed in ion-beam-sputtered fully amorphous alloy Fe_73.9_Cu_0.9_Nb_3.1_Si_13.2_B_8.9_ films upon irradiation with 120 MeV ^108^Ag ions [64]. The magneto-optic Kerr effect (MOKE) indicated a gradual decrease in the magnetic anisotropy with the fluence in these samples irradiated with 350 MeV ^197^Au ions [61]. With the help of HRTEM, the formation of nanocrystalline iron boride phases was observed in an amorphous Fe_73.5_Cu_1_Nb_3_Si_13.5_B_9_ alloy irradiated at low temperature with 5 GeV ^207^Pb ions with fluences of 10^11^ ions cm^−2^. The partial crystallization was found to be less pronounced for intermediate and high (3 × 10^13^ ions cm^−2^) fluences than for low fluence [65,66]. On the other hand, no crystallization at all was observed when a acceptable thermal contact between the irradiated sample and the sample holder was assured in the amorphous and nanocrystalline NANOPERM-type Fe_75_Mo_8_Cu_1_B_16_ alloy irradiated by 130 keV ^14^N^+^ ions at fluences up to 2.5 × 10^17^ ions cm^−2^ [63]. For rapidly quenched Fe_74_Cu_1_Nb_3_Si_16_B_6_ amorphous alloys, being in as-quenched state and irradiated by 593 MeV ^197^Au ions, a dose-dependent decrease in the relative areas of the second and fifth lines of the sextets in the Mössbauer spectra was observed [62].

In the previous work of [47], as-quenched and stress-field-annealed FINEMET ribbons were irradiated by 246 MeV ^84^Kr, 470 MeV ^132^Xe, and 720 MeV ^209^Bi ions and investigated using ^57^Fe Mössbauer spectroscopy and XRD methods. The change in the relative areas of the second and fifth lines of the sextets indicated significant changes in the magnetic anisotropy in the bulk of both as-quenched and stress-annealed FINEMET upon irradiation by swift heavy ions. Differences were observed between the effect of irradiation by various ions having different energies and fluences. The effect of irradiation on the magnetic orientation in FINEMET was explained in terms of radiation-induced defects.

In the present research, we aimed at studying differently stress-field-annealed FINEMET samples, irradiated by swift heavy ions, to determine the change in the magnetic anisotropy due to irradiation both in the bulk and at the surface. A comparison of the radiation effect at the surface and in the bulk is of interest since structural differences can be expected between them due to the different solidification of the amorphous phase. This may alter the effects of the annealing procedure and irradiation as well. We applied ^57^Fe Mössbauer spectroscopy as the main investigating method, which is a unique tool to determine the direction of magnetization in FINEMET alloys. Furthermore, the transmission Mössbauer spectra provide information about the bulk of the ribbon, while the conversion electron Mössbauer spectra (CEMS) can monitor the subsurface in a depth of about 100 nm. We also used X-ray diffractometry to investigate whether the crystal structure of the FINEMET samples can or cannot change upon high-energy heavy ion irradiation.

## 2. Materials and Methods

The Fe_73.5_Si_13.5_Nb_3_B_9_Cu_1_ amorphous alloy was prepared in the form of a 1 cm wide and 20 μm thick ribbon using the standard procedure of rapid quenching of the melt on a rotating disc (melt spinning method). Then, stress annealing of ribbons was performed in a longitudinal furnace at 550 °C for 1 h in Ar atmosphere under different levels of longitudinal tensile stress. The levels of longitudinal stress were applied by weights placed on the ribbon samples. The different stress annealing conditions resulted in samples with different relative permeabilities. Permeability (*μ*) of the samples was measured using an impedance meter. The samples labeled CK021 with *μ* = 2000 and CK022 with *μ* = 8000 as well as CK023 with *μ* = 190,000 were subjected to swift heavy ion irradiation and ^57^Fe Mössbauer spectroscopy as well as XRD measurements. 

Swift heavy ion irradiation of FINEMET ribbons was carried out by 160 MeV energy ^132^Xe^8+^ ions at a fluence of 1 × 10^13^ ion cm^−2^ at room temperature, at a current density of 0.01 μA cm^−2^, and at a pressure of about 10^−3^ Pa, in the IC-100 cyclotron of the Flerov Laboratory of Nuclear Reactions, JINR, Dubna, Russian Federation. In the case of the first irradiation, the wheel side (the side that was in contact with the rotating wheel during rapid quenching) of the stress-annealed FINEMET ribbons (CK021 and CK022) was placed onto a Cu target backed with double-sided carbon tape. After characterization of these samples, for the second irradiation, the carbon tape was fully removed, and irradiation was performed from the air side direction. The ribbon plane of samples was oriented perpendicular to the direction of the ion beam. The temperature of the target backing was controlled during the irradiation.

^57^Fe Mössbauer spectroscopy measurements of non-irradiated and irradiated FINEMET samples were carried out both in transmission and reflection geometry at room temperature. In transmission geometry, the whole volume (i.e., bulk) of the sample can be monitored, while in reflection geometry, using the detection of conversion electrons, only the very thin surface layer (~100 nm) can be studied. Both the transmission and the CEMS spectra of the ribbons were recorded with conventional Mössbauer spectrometers (WISSEL type), working in constant acceleration mode using integrated multichannel analyzers and collecting counts in 512 channels. The transmission Mössbauer spectra were taken using a scintillation detector with a ^57^Co(Rh) source of 1.8 GBq activity, while CEMS measurements used a flowing gas (96% He, 4% CH_4_) proportional counter CEMS detector (RANGER type) and a ^57^Co(Rh) source of 0.8 GBq activity. For all Mössbauer measurements, the direction of the γ-rays was perpendicular to the ribbon plane of FINEMET samples. The CEMS measurements were performed on the air side of the ribbons (the side that was in contact with the surrounding atmosphere during the rapid quenching process of the preparation). Isomer shifts are given relative to α-iron. The evaluation of Mössbauer spectra was performed using least-square fitting of the lines using the MOSSWINN code [67].

For comparison, selected room temperature Mössbauer spectra of non-irradiated ribbons were also recorded in 4096 channels, with a significantly lower instrumental error, using an automated precision Mössbauer spectrometric system developed on the base of the spectrometer SM-2201 with a high velocity resolution, i.e., with the velocity reference signal formed by a digital-analog converter using discretization of 2^12^ (details in [68,69,70]). The spectra were measured in the transmission geometry with a moving absorber using a ^57^Co(Rh) source with activity of 1.8 GBq and a scintillation detector with a NaI(Tl) crystal of 0.1 mm thickness.

Powder X-ray diffractograms of the samples were measured with a computer controlled DRON-2 X-ray diffractometer using CoK_α_ radiation (*λ* = 0.179026 nm) and β filter at room temperature in Bragg–Brentano geometry. This way of recording of the powder XRD patterns was chosen for the easiest identification of the crystalline phases occurring in the investigated samples by comparison with our previous XRD measurements and standard literature data obtained the same way. The XRD diffractograms were recorded at tube generation of 45 kV and 35 mA in the range of 2*Θ* = 20–90° with a goniometer speed of 0.25° min^−1^. The evaluation of the XRD patterns was made using the EXRAY code. For identification of the phases, the PCPDF diffraction data were used.

## 3. Results and Discussion

**Figure 1:**
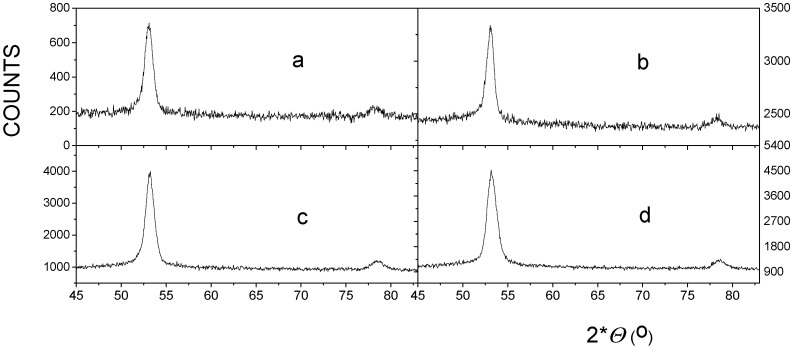
XRDs of FINEMET samples CK021 (**a**,**c**) and CK022 (**b**,**d**), non-irradiated (**a**,**b**) and irradiated by 160 MeV ^132^Xe ions at a fluence of 10^13^ ion cm^−2^ (**c**,**d**).

Figure 1 shows the X-ray diffractograms of the FINEMET samples of CK021 and CK022 before and after irradiation with 160 MeV ^132^Xe ions with a fluence of 10^13^ ion cm^−2^.

All diffractograms show two main peaks, which can be readily associated with (220) and (400) reflections of nanocrystalline Fe-Si phase formed after the heat treatment of the FINEMET ribbon at 550 °C for 1 hour. The lines were centered at 2*Θ* = 53.0° for (220) and 2*Θ* = 78.3° for (400) reflections, which resulted in a lattice parameter of 0.5663 nm in an excellent agreement with the ASTM card of 47-1114 in the PCPDF diffraction data and with our earlier results [47,48] obtained with FINEMET alloys prepared under similar conditions. 

No new peaks appeared and no shifts in the peak positions within the experimental errors were observed in the XRD patterns of the FINEMET samples CK021 (*μ* = 2000) and CK022 (*μ* = 8000) prepared under different stress annealing conditions and those after the irradiation. This indicates that the applied changes in stress annealing do not influence the nanocrystalline structure of the FINEMET ribbons in positive agreement with our previous results [48]. More importantly, this shows that swift heavy iron irradiation by 160 MeV ^132^Xe ions at a fluence of 10^13^ ion cm^−2^ does not induce new phases or further transformation in the already existing nanocrystalline phase. This result is consistent with earlier results by Kuzmann et al. [47] and Miglierini et al. [62], where annealed FINEMET alloys were subjected to swift heavy ion irradiations. This can also demonstrate the favorable thermal contact between the irradiated sample and the target backing, i.e., no heat effect of irradiation can be detected in our case.

Furthermore, we have observed no change in the relative intensities of the corresponding XRD lines within the experimental errors before and after swift heavy ion irradiation. This reflects that no change occurred in the geometrical texture of the FINEMET ribbons upon irradiation by 160 MeV ^132^Xe ions at a fluence of 10^13^ ion cm^−2^. Our present XRD results are in positive agreement with our earlier ones [47], which showed no changes in crystallinity, phase composition, and texture of samples due to swift heavy ion irradiation. 

**Figure 2:**
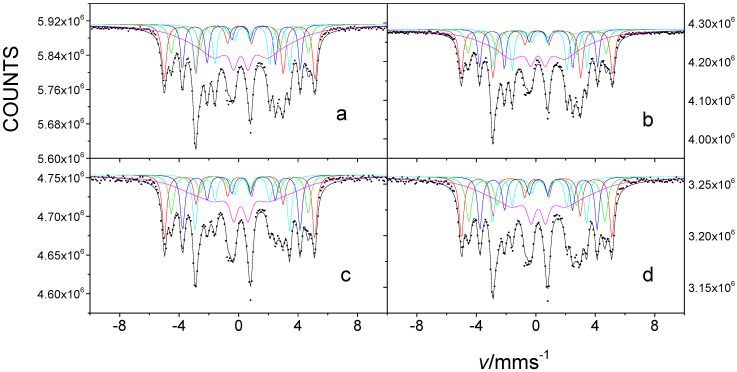
Room temperature transmission ^57^Fe Mössbauer spectra of FINEMET samples CK021 (**a**,**c**) and CK022 (**b**,**d**), non-irradiated (**a**,**b**) and after their first irradiation by 160 MeV ^132^Xe ions at a fluence of 10^13^ ion cm^−2^ (**c**,**d**) from the air side direction.

Figure 2 shows transmission ^57^Fe Mössbauer spectra of FINEMET samples of CK021 and CK022 before and after irradiation by 160 MeV ^132^Xe ions at a fluence of 10^13^ ion cm^−2^. All Mössbauer spectra, depicted in Figure 2, could be satisfactorily decomposed into five sextets corresponding to the different iron microenvironments occurring either in the nanocrystalline Fe-Si or in the amorphous phase. Three sextets are assigned to iron atoms in sublattice A with four, five, and six iron nearest neighbors, one sextet belongs to iron either in sublattice D and/or in sublattice A with seven and eight iron nearest neighbors in Fe-Si, and one sextet is associated with the amorphous component [45]. This method of evaluating the FINEMET spectra is the same as that successfully applied in earlier works [47,48]. 

**Figure 3:**
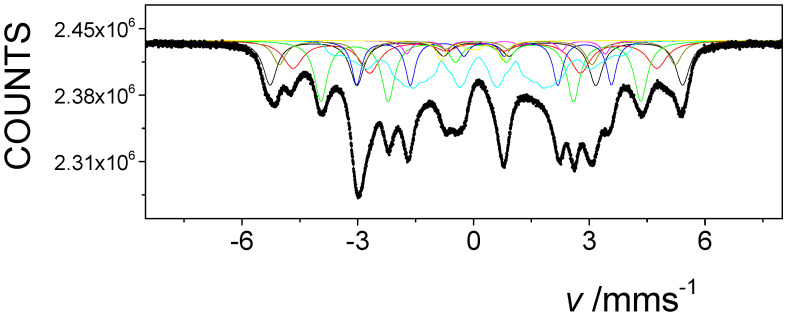
Room temperature ^57^Fe Mössbauer spectrum of non-irradiated FINEMET sample of CK022, recorded with the high velocity resolution Mössbauer spectrometer in 4096 channels in transmission geometry.

The evaluation of the Mössbauer spectra, recorded with a high velocity resolution spectrometer, and of selected non-irradiated FINEMET samples confirmed this type of spectrum evaluation (e.g., Figure 3). This spectral decomposition is especially advantageous for detecting the changes in the magnetic anisotropy of FINEMET samples, as was shown in the previous cases upon stress-induced annealing [48,49] and/or upon irradiation by swift heavy ions [47].

The magnetic hyperfine field and isomer shift values, which were obtained for the spectra in Figure 2 and that are characteristic of the individual sextet components (see in Supplementary Materials, Table S1), are in positive accordance with those reported earlier [47,48]. They were found to be practically the same for the corresponding sextets both for the stress-annealed and the irradiated cases. This means that, besides the considerable changes found in the magnetic anisotropy, no other significant changes in the local iron microenvironments can be observed at the different stress annealing conditions and at the irradiation.

From the perspective of our present goal, the most important result is that we are able to obtain quantitative information about the magnetic anisotropy occurring in the studied FINEMET samples through the evaluation of Mössbauer spectra. By applying the aforementioned evaluation of the spectra, we determined the relative areas of the sextet spectral lines using the MOSSWINN code [67]. The area ratio *A*_2,5_/*A*_1,6_ was constrained to be the same for all sextets, while *A*_1,6_/*A*_3,4_ was kept at three. 

According to the Wigner–Eckhart theorem [71], the angular dependence of the relative peak areas of a magnetic sextet appearing in the ^57^Fe Mössbauer spectrum, representing the *I* = 3/2 to *I* = 1/2 nuclear spin transition, can be derived [41] when, apart from a constant factor, one obtains *A*_1_(*θ*) = *A*_6_(*θ*) = 3 (1 + cos^2^*θ*) and *A*_2_(*θ*) = *A*_5_(*θ*) = 4 sin^2^*θ* where *θ* is the angle included by the respective directions of the γ-rays and the effective magnetic field and the area subscripts refer to the peak numbers of the sextet. In the case of magnetic anisotropy, *A*_2,5_ compared to *A*_1,6_ depends on *θ,* as was shown by Equation (1).

By applying Equation (1) we calculated the angle *θ* included by the directions of magnetization and γ-rays using the ratio of *A*_2,5_/*A*_1,6_ deduced from the evaluations of the Mössbauer spectra of the FINEMET samples, similarly to that reported in the previous works [47,48,58]. The values of *A*_2,5_/A_1,6_ and *θ*, which we obtained for FINEMET samples CK021 and CK022, non-irradiated and irradiated by 160 MeV ^132^Xe ions at a fluence of 10^13^ ion cm^−2^, respectively (Figure 2), are shown in Table 1.

The different *θ* values shown in Table 1 reflect different orientations of magnetization for all the investigated FINEMET samples. This can be associated with changes in the magnetic anisotropy either with the different stress annealing conditions in FINEMET samples of different permeabilities or upon irradiation. 

By considering that the direction of magnetization is parallel to the plane of the FINEMET ribbon without any (heat or irradiation) treatment when *A*_2,5_/A_1,6_ = 4 and *θ* = 90° [48], we can measure the changes in magnetic anisotropy compared to the ribbon plane direction. Since the direction of γ-rays is perpendicular to the ribbon plane, *θ* is the complementary angle to that characteristic of changes in magnetic anisotropy for annealing or for irradiation, as was mentioned previously [47,48].

The different *θ* angles obtained with non-irradiated samples of different permeabilities, annealed with different tensile weights (62.5° and 70.3° for samples with *μ* = 2000 and *μ* = 8000, respectively), show that the average direction of magnetization differs due to stress annealing, i.e., the degree of stress annealing induced by magnetic anisotropy depends on the relative permeability of the sample, and consequently on the applied tensile weight (the higher weight in producing the stress field leads to the lower relative permeability [48]). 

More importantly, from the point of view of the goal of our present research, we have observed significant changes in the orientation of magnetization of the FINEMET samples due to the first irradiation (applied from the direction of the air side of the ribbons) by 160 MeV ^132^Xe ions at a fluence of 10^13^ ion cm^−2^. Namely, as Table 1 shows, the angle included by the directions of magnetization and γ-rays changed from 62.5° to 45.4° and from 70.3° to 53.3° for samples with *μ* = 2000 and *μ* = 8000, respectively, after the irradiation. This clearly evidences the decrease in the magnetic anisotropy in the FINEMET samples as the effect of the applied swift heavy ion irradiation.

This is in agreement with the results found earlier for the effect of irradiation in FINEMET or related alloys [47,58,62] and confirms that swift heavy ion irradiation induces considerable changes of magnetic anisotropy not only in the amorphous phase but in the Fe-Si nanocrystallites as well. However, the values of the orientation angle of magnetization after irradiation and the values of the change in magnetization were found to be different from those experienced in the earlier work [47] in stress-annealed samples upon irradiation by the swift heavy ions of ^209^Bi, ^132^Xe and ^84^Kr. These differences may be understood in the different conditions of irradiation (energy and fluence of the ions) and of the sample preparation. 

Another interesting result, which can be derived from the analysis of the data depicted in Table 1, is that the same change in magnetic anisotropy (~17° difference in the direction of magnetization) can be observed before and after irradiation by 160 MeV ^132^Xe ions at a fluence of 10^13^ ion cm^−2^ in both samples, independently from the direction of magnetization in the non-irradiated state. Similar changes were experienced when 720 MeV ^209^Bi ion irradiation at a fluence of 5 × 10^11^ ion cm^−2^ was applied on FINEMET samples [47].

However, we should take into account that only about a half of the volume of the ribbons could be irradiated by the presently applied swift heavy ion irradiation according to the estimation by the SRIM-13 code [72] of energy deposition of ions in the FINEMET ribbons. To irradiate the whole volume, the first-time irradiated samples were irradiated for the second time from the opposite direction (from the wheel side direction) of the ribbons.

**Figure 4:**
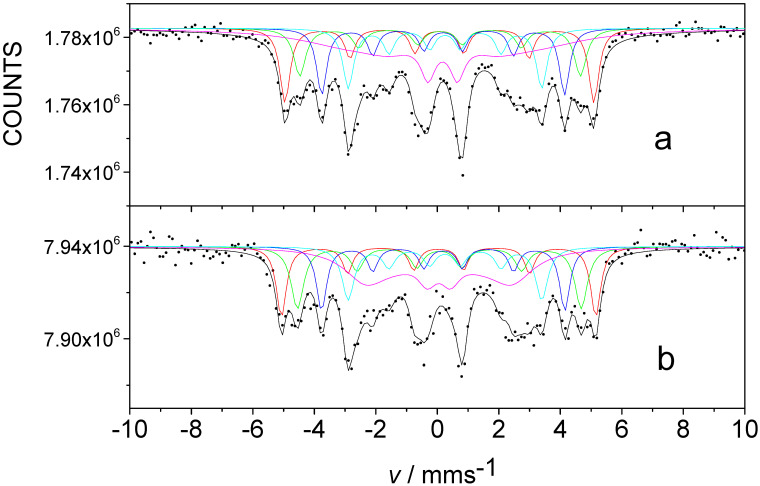
Room temperature transmission ^57^Fe Mössbauer spectra of FINEMET samples CK021 (**a**) and CK022 (**b**) after their second irradiation by 160 MeV ^132^Xe ions at a fluence of 10^13^ ion cm^−2^ when they were irradiated from both sides.

From the data obtained, it can be concluded that the CK021 and CK022 FINEMET magnetic orientation (Table 1) irradiated by ^132^Xe ions with an energy of 160 MeV from both sides (Figure 4 and Table 1) is significantly smaller for both samples than for the samples irradiated from one side (Figure 2 and Table 1) and even smaller than that of the non-irradiated sample (Figure 2, Table 1). It is clear from this that swift heavy ion irradiation applied from both sides further changes the average orientation of the magnetic moments compared to that of the magnetic orientation caused by the single-sided irradiation, in a way that the magnetic anisotropy is further reduced. Furthermore, it can be observed that the effect of the second irradiation on both samples changed the magnetic orientation to a lesser extent than that for the first irradiation. Compared to the first irradiation examined, the CK021 sample with lower permeability irradiated on both sides showed a smaller change in irradiation from the other side (*A*_2,5_/*A*_3,4_ = from 1.36 to *A*_2,5_/*A*_3,4_ = 1.14), while the higher permeability sample CK022 (both sides irradiated), starting from higher area ratios (*A*_2,5_/*A*_3,4_ = 1.9), exhibited a change from *A*_2,5_/*A*_3,4_ = 1.9 to *A*_2,5_/*A*_3,4_ = 1.20. From this, it can be concluded that the effect of irradiation on magnetic anisotropy can be influenced by the initial state of magnetic anisotropy. If the effect of the first irradiation on the orientation angle *θ* is extrapolated to a linear approximation for the second irradiation, the second irradiation would result in a higher change in the orientation angle than that experienced. The fact that this did not occur suggests that there is a boundary angle (near the alternate angle of the magic angle) for the change in the direction of the magnetic anisotropy due to high-energy heavy ion irradiation at which the magnetic anisotropy is at a minimum. This conclusion is in positive agreement with the fact that the effect of irradiation of FINEMET samples with Bi and Kr ions [47] with much higher energy than in the case of the present irradiation resulted in an orientation angle not lower than the mentioned limit. An important result is that by learning about the results of irradiation of the above-mentioned FINEMET alloys on both sides, it was possible to obtain information about the change in the anisotropy of the samples compared to the non-irradiated samples in the total sample volume. Total irradiation in both samples led to a change in anisotropy near the cut-off angle. Comparing with the previous literature data [47], we can conclude that ^132^Xe irradiation with an energy of 160 MeV and a dose of 10^13^ ions cm^−2^ can have a near-maximum effect on the orientation of magnetization.

This result is in positive agreement, completing the previous results obtained for FINEMET samples produced under different conditions [48] and supporting the validity of the correlations obtained there.

**Figure 5:**
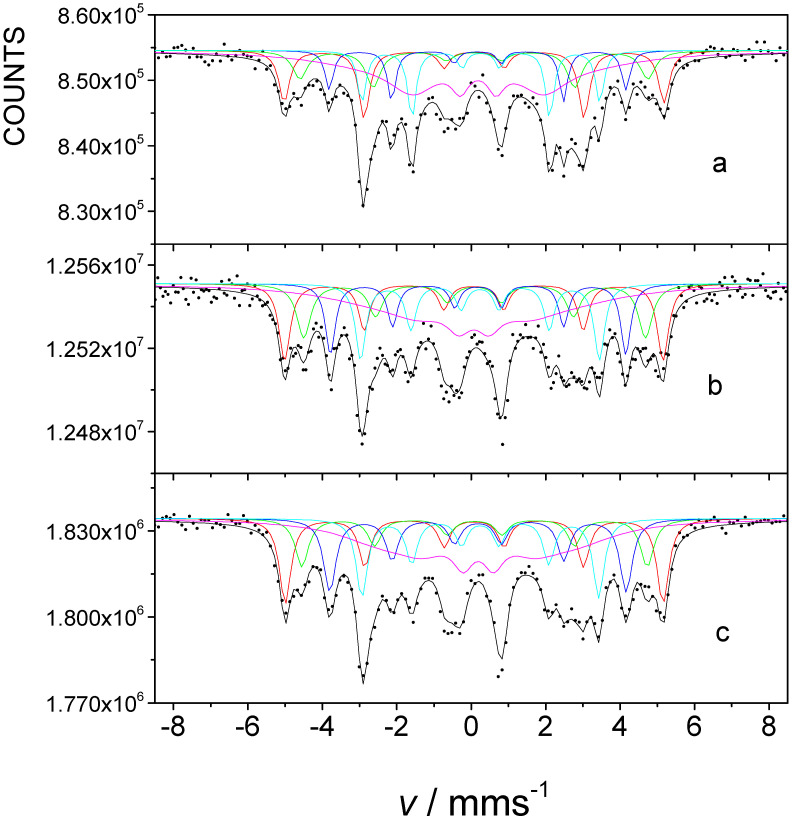
Room temperature transmission ^57^Fe Mössbauer spectra of FINEMET samples CK023 (**a**) non-irradiated and after their first irradiation by 160 MeV ^132^Xe^28+^ ions at a fluence of 10^13^ ion cm^−2^ from the air side (**b**) and from the wheel side (**c**).

The FINEMET alloy with extremely high permeability (*µ* = 190,000) is of particular importance for technical applications. Consistent with the high permeability, based on previous work [48], the orientation of the magnetic moment near the plane of the sample is expected to be the dominant effect of the shape anisotropy. Accordingly, it is particularly interesting to what extent the high-energy heavy ion irradiation we use can cause an anisotropy change in this alloy. On the other hand, we also examine whether there is a difference when irradiation from one side is applied, from the wheel side or from the air side. To study all of this, in addition to the non-irradiated sample with a permeability of 190,000, we examined the effect of irradiation by ^132^Xe ions with an energy of 160 MeV at two different times on two separate pieces of the sample using transmission Mössbauer spectroscopy. Figure 5a shows the room temperature Mössbauer spectrum of a non-irradiated CK023 sample with a permeability of 190,000. The data for the measurement conditions are summarized in Table 2.

The angle *θ* calculated from *A*_2,5_/*A*_3,4_ = 3.9 is 83.54°. Indeed, this shows that the magnetic moments are almost parallel with the plane of the sample, in accordance with said expectation and the correlation between permeability and magnetic anisotropy [48].

Already, from the comparison of the Mössbauer spectra shown in Figure 5a–c and from Table 2, the change in the relative area of lines two and five, i.e., the change in magnetic anisotropy due to swift heavy ion irradiation, is evident. The angle *θ* calculated from the data of *A*_2,5/_*A*_3,4_ in Table 2 is 51.98°.

The resulting transmission Mössbauer spectra are similar to those previously evaluated for the cases of CK021 and CK022 samples. Swift heavy ion irradiation resulted in a significant decrease in relative line area ratios, thereby indicating a reduction in the angle included by the γ-radiation and the magnetic moments. This is consistent with previous observations [47]. The change in the orientation of the magnetic moment of the non-irradiated sample with *µ* = 190,000 permeability due to irradiation is much larger than that observed with the much lower permeability samples CK021 and CK022.

We note that one of the pristine non-irradiated samples after irradiation from the air side has an angle of 51.98°, included by the γ-radiation and magnetic moments, while another non-irradiated sample irradiated from the wheel side almost a year later but under the same conditions showed this value to be 51.13° (Table 2). The difference is within the standard deviation. This indicates reproducibility of irradiation and the applicability of the Mössbauer method for these investigations.

**Figure 6:**
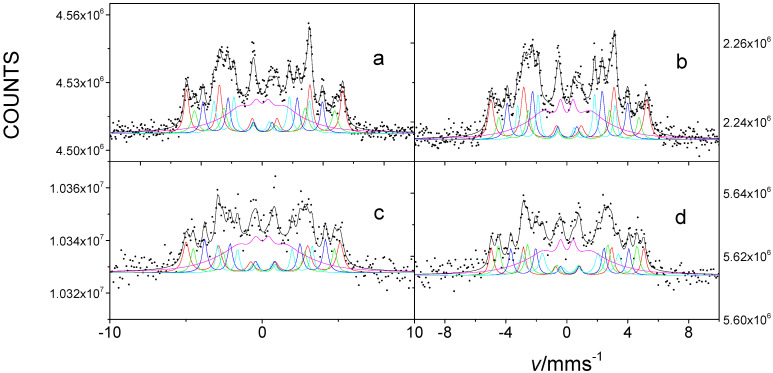
Room temperature ^57^Fe conversion electron Mössbauer spectra of FINEMET samples CK021 (**a**,**c**) and CK022 (**b**,**d**), non-irradiated (**a**,**b**) and irradiated with 160 MeV ^132^Xe ions with a fluence of 10^13^ ion cm^−2^ (**c**,**d**).

Figure 6 shows ^57^Fe CEMS spectra of FINEMET samples CK021 and CK022 before and after irradiation by 160 MeV ^132^Xe ions at a fluence of 10^13^ ion cm^−2^. The conversion electron Mössbauer spectra were successfully decomposed into five sextets belonging to the different microenvironments of Fe in nanocrystalline Fe-Si phase and in amorphous matrix, similarly as in the case of the evaluation of transmission Mössbauer spectra (Figure 2). The isomer shifts and magnetic hyperfine fields of the individual components were also in positive agreement with those obtained from the transmission Mössbauer measurements. This can reflect that the surface (within ~100 nm) of the FINEMET samples consists of nanocrystalline Fe-Si particles embedded in amorphous matrix, similarly to that of the bulk.

We found significant changes comparing the CEMS spectra and the derived parameters characterizing the direction of magnetization both in the case of differently stress-annealed non-irradiated samples as well as before and after their irradiation by 160 MeV ^132^Xe ions at a fluence of 10^13^ ion cm^−2^. The values of *A*_2,5_/*A*_1,6_ and *θ* that we obtained for FINEMET samples CK021 and CK022 from their CEMS spectra are shown in Table 3.

It can be clearly seen in Table 3 that different stress annealing conditions result in different *θ* values (86.8° and 74.5° for samples with *μ* = 2000 and *μ* = 8000, respectively) at the surface of the FINEMET ribbon. The tendency of changes in the direction of magnetization as a function of permeability is similar to that obtained in the bulk and was established for the bulk earlier [48]. However, these *θ* values are higher than those that are characteristic of the bulk (compare the corresponding data of Table 1, Table 2 and Table 3), which reflect that higher anisotropy can be induced by stress annealing in the bulk compared to that at the surface. This can be explained by the fact that, at the surface (where the spins have a strong tendency to be oriented parallel to the plane of the ribbon), the shape anisotropy effect is much larger than in the bulk. Furthermore, the higher deflection of the spins from the plane at the surface as compared to that in the bulk, for the sample with lower permeability presumably needs a different explanation, e.g., related to fluctuations in the defect structure.

More importantly, we have found that the magnetic anisotropy decreases at the surface of FINEMET ribbons due to swift heavy ion irradiation. Namely, as Table 3 shows, the angle *θ* included by the directions of magnetization and γ-rays was changed after irradiation from 74.4° to 62.9° and from 86.8° to 69.2° for samples with *μ* = 2000 and *μ* = 8000, respectively. This effect that we observed at the surface of FINEMET ribbons at present is in positive agreement with radiation-induced anisotropy changes found in the bulk FINEMET [47], as well as in the bulk [56] and at the surface [60] of soft magnetic amorphous alloys and also in thin crystalline films [73,74,75].

However, we have observed that the final directions of magnetization at the surface of FINEMET ribbons after irradiation (62.9° and 69.2° for samples with *μ* = 2000 and *μ* = 8000, respectively) are different from those (45.4° and 53.3°) that occurred in the bulk. Furthermore, the change in magnetic anisotropy induced by irradiation, i.e., the difference in the angle of the direction of magnetization before and after irradiation, depends on the annealing history of the sample (~12° for sample with *μ* = 2000 and ~17° for sample with *μ* = 8000). By comparing the irradiation effects between the surface and the bulk, we can establish that the degree of induced anisotropy change is higher in the bulk than at the surface for the sample with *μ* = 2000, and they are comparable for the sample with *μ* = 8000. This can indicate that the radiation-induced anisotropy changes depend on the initial anisotropy much more at the surface than in the bulk.

Our results can be understood in the framework of the mechanism of irradiation-induced defects proposed previously for the explanation of anisotropy changes induced by swift heavy ion irradiation in amorphous and nanocrystalline alloys [47,58,61]. Accordingly, our findings can be associated with the defects induced by irradiation when the stress with anisotropic distribution, occurring around these defects, modify the orientation of spins depending on the direction of the stress. On the other hand, some contribution from spin reorientation around the stress centers formed during the inhomogeneous solidification, mainly in the amorphous matrix or at the surface, should be taken into consideration as a consequence of mixing atoms due to irradiation. 

This can be supported by the data of the electronic stopping power (*dE_e_/dx*) of 3.06 × 10^4^ keV μm^−1^, nuclear stopping power (*dE_n_/dx*) of 151.7 keV μm^−1^, and vacancy/ion ratio of 61,425, calculated with the SRIM-13 code [72] for the presently applied 160 MeV ^132^Xe ion irradiation on FINEMET samples. The deviation of these data from those obtained for 470 MeV ^132^Xe ion irradiation used earlier [47] has a favorable correlation with the lower degree of the induced anisotropy obtained at present than that in the case of 470 MeV ^132^Xe ion irradiation [47]. The irradiation-induced magnetic anisotropy was found to be higher or comparable in the bulk than that at the surface. The electronic excitation is the dominant interaction for inducing change in the direction of magnetization of FINEMET samples due to swift heavy ion irradiation. This can also be supported by the XRD data; no irradiation-induced crystallization could be observed in the irradiated bulk.

## 4. Conclusions

The following conclusions can be drawn from the significant changes in the relative areas of transmission and conversion electron Mössbauer lines of the differently stress-annealed Fe_73.5_Si_13.5_Nb_3_B_9_Cu_1_ FINEMET ribbons irradiated by 160 MeV ^132^Xe ions compared to those of non-irradiated ones:

- Swift heavy ion irradiation induces considerable changes in the orientation of magnetization (relative to the normal of the ribbon plane), i.e., a decrease in the magnetic anisotropy, both in the bulk and at the surface of the FINEMET ribbons;
- The orientation of magnetization after irradiation is significantly different in the bulk and at the surface. The direction of magnetization is closer to the ribbon plane at the surface than that in the bulk;
- The orientation of magnetization after irradiation depends on the initial magnetic anisotropy produced by stress annealing. The stress-annealing-caused anisotropy is lower at the surface than that in the bulk;
- The decrease in the magnetic anisotropy due to swift heavy ion irradiation can be mostly associated with a change in spin orientation around the radiation-induced defects;
- The present findings for the bulk complement support the previous results [47];
- Swift heavy ion irradiation can be a useful tool for fine tuning the magnetic anisotropy in FINEMET alloys to achieve optimal soft magnetic properties.

## Figures and Tables

**Table 1:** Effect of swift heavy ion irradiation on the orientation of magnetization in the bulk of differently stress-annealed FINEMET ribbons.

Sample	*A*_2,5_/*A*_1,6_	*θ* (°)
***CK021***		
non-irradiated	2.59	62.5
*µ* = 2000		
***CK021***		
first irradiated from the air side		
160 MeV ^132^Xe, 1 × 10^13^ ion cm^−2^	1.36	45.4
***CK021***		
second irradiated from the wheel side		
(both side irradiated)		
160 MeV ^132^Xe, 1 × 10^13^ ion cm^−2^	1.15	41.93
***CK022***		
non-irradiated	3.18	70.3
*µ* = 8000		
***CK022***		
first irradiated from the air side		
160 MeV ^132^Xe, 1 × 10^13^ ion cm^−2^	1.89	53.3
***CK022***		
second irradiated from the wheel side		
(both side irradiated)		
160 MeV ^132^Xe, 1 × 10^13^ ion cm^−2^	1.2	42.79

*A*_2,5_/*A*_1,6_ is the ratio between the relative areas of the second and fifth lines and the first and sixth lines, and angle *θ* is the angle included by the directions of magnetization and γ-rays. The data were obtained from the evaluation of the corresponding transmission Mössbauer spectra. The standard deviation of *θ* is ±0.15°.

**Table 2:** Effect of swift heavy ion irradiation on the orientation of magnetization in the bulk FINEMET ribbons with high permeability.

Sample	*A*_2,5_/*A*_1,6_	*θ* (°)
***CK023***		
non-irradiated	3.9	83.54
*µ* = 190,000		
***CK023***		
first irradiated from the air side		
160 MeV ^132^Xe, 1 × 10^13^ ion cm^−2^	1.8	51.98
***CK023***		
first irradiated from the wheel side		
160 MeV ^132^Xe, 1 × 10^13^ ion cm^−2^	1.74	51.13

*A*_2,5_/*A*_1,6_ is the ratio between the relative areas of the second and fifth lines and the first and sixth lines, and angle *θ* is the angle included by the directions of magnetization and γ-rays. The data were obtained from the evaluation of the corresponding transmission Mössbauer spectra. The standard deviation of *θ* is ±0.15°.

**Table 3:** Effect of swift heavy ion irradiation on the orientation of magnetization at the surface of differently stress-annealed FINEMET ribbons.

Sample	*A*_2,5_/*A*_1,6_	*θ* (°)
***CK021***		
non-irradiated	3.46	74.4
*µ* = 2000		
***CK021***		
irradiated		
160 MeV ^132^Xe, 1 × 10^13^ ion cm^−2^	2.62	62.9
***CK022***		
non-irradiated	3.97	86.8
*µ* = 8000		
***CK022***		
irradiated		
160 MeV ^132^Xe, 1 × 10^13^ ion cm^−2^	3.1	69.2

*A*_2,5_/*A*_1,6_ is the ratio between the relative areas of the second and fifth lines and the first and sixth lines, and angle *θ* is the angle included by the directions of magnetization and γ-rays. The data were obtained from the evaluation of the corresponding conversion electron Mössbauer spectra. The standard deviation of *θ* is ±0.2°.

## Data Availability

Relevant data have been shown in the paper.

## Supplementary Materials

The following supporting information can be downloaded at: https://www.mdpi.com/article/10.3390/nano12121962/s1, Table S1: Mössbauer hyperfine parameters of spectral components (shown in Figure 2) of FINEMET samples.
