# Supplementary material for: Change in Magnetic Anisotropy at the Surface and in the Bulk of FINEMET Induced by Swift Heavy Ion Irradiation
Source: Nanomaterials (Basel). 2022 Jun 8;12(12):1962. doi: 10.3390/nano12121962 (PMC9228724; doi:10.3390/nano12121962)
Supplement: Supplementary file 1 [file nanomaterials-12-01962-s001.zip › nanomaterials-1758076-supplementary.pdf]

Supplementary Materials

Change in Magnetic Anisotropy at the Surface and in the Bulk of FINEMET Induced by Swift Heavy Ion Irradiation

Table S1. Mössbauer hyperfine parameters of spectral components (shown in Figure 2) of FINEMET samples.

		**Sample**			
**Sextet**	**parameter**	**Non-irradiated CK021**	**Irradiated with 160 MeV ^{132}Xe, 1×10^{13} ion cm^{-2} CK021**	**Non-irradiated CK022**	**Irradiated with 160 MeV ^{132}Xe, 1×10^{13} ion cm^{-2} CK022**
S1	δ [mm s^{-1}]	0.07 (±0.01)	0.07 (±0.01)	0.07 (±0.01)	0.06 (±0.01)
	B [T]	31.7 (±0.1)	31.7 (±0.1)	31.5 (±0.1)	31.5 (±0.1)
S2	δ [mm s^{-1}]	0.08 (±0.01)	0.08 (±0.01)	0.08 (±0.01)	0.07 (±0.01)
	B [T]	28.8 (±0.1)	28.8 (±0.1)	28.6 (±0.1)	28.6 (±0.1)
S3	δ [mm s^{-1}]	0.18 (±0.01)	0.18 (±0.01)	0.18 (±0.01)	0.18 (±0.01)
	B [T]	24.7 (±0.1)	24.7 (±0.1)	24.6 (±0.1)	24.6 (±0.1)
S4	δ [mm s^{-1}]	0.25 (±0.01)	0.25 (±0.01)	0.25 (±0.01)	0.25 (±0.01)
	B [T]	19.7 (±0.1)	19.7 (±0.1)	19.7 (±0.1)	19.7 (±0.1)
S5	δ [mm s^{-1}]	0.14 (±0.02)	0.14 (±0.02)	0.15 (±0.02)	0.15 (±0.02)
	B [T]	18.9 (±0.2)	18.9 (±0.2)	18.9 (±0.2)	18.9 (±0.2)
